# Characterization and Heterologous Expression of UDP-Glucose 4-Epimerase From a *Hericium erinaceus* Mutant with High Polysaccharide Production

**DOI:** 10.3389/fbioe.2021.796278

**Published:** 2021-11-25

**Authors:** Gen Zou, Juanbao Ren, Di Wu, Henan Zhang, Ming Gong, Wen Li, Jingsong Zhang, Yan Yang

**Affiliations:** ^1^ National Engineering Research Center of Edible Fungi, Institute of Edible Fungi, Shanghai Academy of Agricultural Sciences, Shanghai, China; ^2^ College of Food Sciences and Technology, Shanghai Ocean University, Shanghai, China

**Keywords:** polysaccharide synthesis, heterologous expression, immune activity, enzymatic properties, polysaccharide, *Hericium erinaceus*

## Abstract

*Hericium erinaceus* is an important medicinal fungus in traditional Chinese medicine because of its polysaccharides and other natural products. Compared terpenoids and polyketides, the analysis of synthetic pathway of polysaccharides is more difficult because of the many genes involved in central metabolism. In previous studies, *A6180*, encoding a putative UDP-glucose 4-epimerase (UGE) in an *H. erinaceus* mutant with high production of active polysaccharides, was significantly upregulated. Since there is no reliable genetic manipulation technology for *H. erinaceus*, we employed *Escherichia coli* and *Saccharomyces cerevisiae* to study the function and activity of A6180. The recombinant overexpression vector pET22b-A6180 was constructed for heterologous expression in *E. coli*. The enzymatic properties of the recombinant protein were investigated. It showed that the recombinant A6180 could strongly convert UDP-α-D-glucose into UDP-α-D-galactose under optimal conditions (pH 6.0, 30°C). In addition, when *A6180* was introduced into *S. cerevisiae* BY4742, xylose was detected in the polysaccharide composition of the yeast transformant. This suggested that the protein coded by *A6180* might be a multifunctional enzyme. The generated polysaccharides with a new composition of sugars showed enhanced macrophage activity *in vitro*. These results indicate that A6180 plays an important role in the structure and activity of polysaccharides. It is a promising strategy for producing polysaccharides with higher activity by introducing *A6180* into polysaccharide-producing mushrooms.

## Introduction

*Hericium erinaceus*, which grows widely in the mountains of eastern Asia, is a valuable fungus for both medicinal and food use and has a long history in the field of medicine in China. Polysaccharides, the main active substance of *H. erinaceus*, have high biological activity, such as immunomodulation (Wu et al., 2019; Nowak et al., 2020), regulation of glucolipid metabolism (Du et al., 2015; Cai et al., 2020), hypolipidemia (Liang et al., 2013), anti -tumor properties (Zeng and Zhu, 2018; Zhang et al., 2021), anti-fatigue properties (Liu et al., 2015), liver protection, and stomach protection (Chen et al., 2020). They have also been widely used in medicine (Li et al., 2018; Jithendra et al., 2020), materials (Low et al., 2015), cosmetics, and other fields. Due to the large number of key enzymes involved in the synthesis of polysaccharides and their unknown characteristics, the synthesis pathway of *H. erinaceus* polysaccharides has not been clearly clarified. In our previous study, two mutant strains with high polysaccharide production of *H. erinaceus* were bred by atmospheric pressure room temperature plasma (ARTP) mutagenesis, and polysaccharide production was significantly enhanced, compared with the original strain (Zhu et al., 2019). Through multi-omics analysis, the increased carbohydrate metabolism and the production of glucose-6-phosphate constituted the basis of high polysaccharide yield. The differentially expressed proteins A6180 involved in the mushroom polysaccharide biosynthetic pathways occurred in the mutant strain compared with the original strain, which belonged to GAL10 (UDP-glucose-4-epimerase) involved in the synthesis of polysaccharide repeat units, and upregulated mRNA and protein expression based on transcriptome and proteomics data (Gong et al., 2021). Upregulation of the A6180 gene is known to be involved in the biosynthesis of polysaccharide repeat units, which is associated with the higher yield of polysaccharides in the mutated strains. However, the function of this gene still needs to be verified through genetic engineering technology.

Using the method of gene overexpression is an important approach to analyze gene function. For example, Jesus et al. constructed recombinant overexpressed strains and homologous overexpressed BL23 gene (encoding UGP) in *Lactobacillus casei*. In the subsequent enzyme activity detection, it was found that the enzyme activity of *L. casei* increased by approximately 70 times, and the concentration of UDP-Glu increased by 8.5 times. Therefore, it is inferred that the BL23 gene is a key gene in the UDP-glucose synthesis pathway (Rodriguez-Diaz and Yebra, 2011).

Since genetic manipulation techniques in most macro fungi are not sophisticated, it is difficult to investigate the function of a gene by knockout or overexpression of a nativegene. Therefore, genetic verification in heterologous or model microorganisms can more effectively detect the functions of target genes (Bachmann et al., 2014). *Escherichia coli* and *Saccharomyces cerevisiae*, as typical eukaryotic model organisms and microbial cell factories, have been widely used in metabolic engineering, system biology, and synthetic biology. For example, *S. cerevisiae* cannot directly use xylan as a carbon source, and strains with high expression can be obtained by integrating xylanase with high enzyme activity into the *S. cerevisiae* genome by means of molecular biology (Lu et al., 2017; Wang et al., 2017). Researchers (Biely et al., 2000) expressed the Xyn2 gene of *Trichoderma reesei* in *S. cerevisiae* using different promoters, *ADH2* and *PGK1*, and the enzyme activity was 200 and 160 nkat/ml. Besides, the *de novo* synthesis pathway of m-cresol was constructed from *S. cerevisiae* glucose by introducing the heterologous pathway of 6-MSA (Hitschler and Boles, 2019). The high yield of emodin reached 528.4 mg/L in *S. cerevisiae* BJ5464-NPGA by heterologous reconstruction of the biosynthesis pathway of endorphin and emodin (Sun et al., 2019).

In this study, *E. coli* and *S. cerevisiae* were used as heterologously expressing chassis organisms to further verify the biological function of the gene *A6180*, which is closely related to high polysaccharide production in *H. erinaceus*. The *A6180* gene clone and different recombinant overexpression vector constructs for heterologous expression will be further studied to find out the functional role of this gene in polysaccharide synthesis. The enzyme properties of the protein expressed by the *A6180* gene were studied to provide a theoretical reference for later development and utilization.

## Materials and Methods

### Strain and Plasmids

The experimental strain used in this study was *H. erinaceus* 321, preserved at the Institute of Edible Mushrooms, Shanghai Academy of Agricultural Sciences. Plasmid pET-22b (+) cells were preserved in our laboratory. *E. coli* strains TOP10 and BL21 (DE3) competent cells were purchased from Weidi Biotechnology (Shanghai, China). *S. cerevisiae* BY4742 was purchased from Zoman Biotechnology (Beijing, China). The pESC-Leu plasmid was purchased from TIANDZ Gene Technology (Beijing, China). To construct the heterologous expression vector, the mycelium of *H. erinaceus* was scraped and total RNA was extracted using TRIzol RNA Isolation Reagents (TAKARA) kit, followed by Hifair TM II 1st Strand cDNA Synthesis Kit (YEASEN, Shanghai, China). The obtained cDNA was used as a template for amplification of the *A6180* coding sequence. Yeast genomic DNA was extracted from cultured *Saccharomyces cerevisiae* BY4742 using the Plant Genomic DNA Extraction Kit (TIANGEN, Beijing, China). The extracted gDNA was used as a template for amplification of the *TDH3* promoter. Primers A6180EC-F and A6180EC-R were designed to amplify the *A6180* gene fragment using Primer Premier 5.0 based on the results of whole genome sequencing of *H. erinaceus* 321, and all the primer sequences used in this study are shown in Table 1. The PCR amplification procedure for the *A6180* target gene was as follows: 3 min at 98°C; 30 cycles of 98°C for 10 s, 58°C for 20 s, 72°C for 80 s; and a final extension at 72°C for 5 min. The pET-22b (+) vector was amplified with restriction endonucleases (QuickCut Nde I and Xho I; TAKARA, Dalian, China). Double digestion was performed, followed by the construction of the recombinant vector (pET22b-A6180) using the Hieff Clone^®^ Plus Multi One Step Cloning Kit (YEASEN) kit.

**TABLE 1:** Primer design of the *Hericium erinaceus A6180* gene and related functional fragment.

Primer	Sequence (5′-3′)	Descriptions
A6180EC-F	AAC​TTT​AAG​AAG​GAG​ATA​TAC​ATA​TGG​CTG​TTG​CCG​ATA​CCT​C	For full-length *A6180*
A6180EC-R	TCA​GTG​GTG​GTG​GTG​GTG​GTG​CTC​GAG​CTT​GGA​CTC​GGT​ATC​GTA​GCC​G	
A6180SC-F	CAC​ACA​TAA​ACA​AAC​AAA​GCG​GCC​GCA​TGG​CTG​TCG​CTG​ATA​CCT​CTC​T	For full-length *A6180*
A6180SC-R	CCT​TGT​AAT​CCA​TCG​ATA​CTA​GTT​CAA​TGA​TGA​TGA​TGA​TGA​TGC​TTC​GAC​TCG​GTA​TCG​TAT​CCA​TTC	
TDH3-F	AAC​CCT​CAC​TAA​AGG​CAT​ATG​ATA​CTA​GCG​TTG​AAT​GTT​AGC​GTC	For full-length *TDH3* promoter
TDH3-R	ATC​AGC​GAC​AGC​CAT​GCG​GCC​GCT​TTG​TTT​GTT​TAT​GTG​TGT​TTA​TTC	

### Expressing *A6180* in *E. coli* BL21

The constructed vector harboring *A6180* (pET22b-A6180) was propagated in TOP10. For heterologous expression of recombinant proteins in *E. coli*, pET22b-A6180 was transformed into *E. coli* BL21 (DE3) competent cells. Positive colonies were screened using ampicillin and designated as BL21-A6180. To obtain sufficient recombinant protein, the optimal induction conditions were tested at different temperatures (15°C and 37°C) with various doses of isopropyl-β-D-thiogalactopyranoside (IPTG) (1.0 and 0.2 mmol/L). The recombinant protein was purified using a His-tagged protein purification kit (Beyotime, Shanghai, China) for assays of enzymatic properties.

### Determination of Enzymatic Properties of Recombinant Protein

High performance liquid chromatography (HPLC) was employed to evaluate the recombinant putative UGE by detecting the conversion rate of UDP-α-D-glucose (UDP-Glu) into UDP-α-D-galactose (UDP-Gal) (Goulard et al., 2001). Thereafter, to study the enzymatic properties of recombinant UGE proteins, such as optimum pH and temperature. In optimal pH assay, different buffers including 10 mM citric acid-sodium citrate buffer (pH 3.0, 4.0, 5.0, and 6.0), 10 mM Tris-HCl buffer (pH 7.0, 8.0 and 9.0), 10 mM sodium carbonate-sodium hydroxide buffer (pH 10.0, and 11.0) were prepared for reaction at 35°C for 1 h. In optimal temperature assay, the enzyme reaction mixture was incubated at different temperatures (15°C, 20°C, 25°C, 30°C, 35°C, 40°C, 45°C, 50°C, and 55°C) for 1 h at optimal pH. In order to determine the effect of different ions on UGE activity, 1.0 mM ions were respectively added into the reaction system, including K^+^, Ni^2+^, Mg^2+^, Ba^2+^, Ca^2+^, Cu^2+^, Co^2+^, and Fe^3+^. The optimal separation conditions were: Athena NH_2_ (250 mm × 4.6 mm, 5 µm); mobile phase: KH_2_PO_4_ buffer (0.125 mol/L, pH 3.6): ethanol = 40:60 (v:v); column temperature: 30°C; flow rate: 1.0 ml/min; detector: UV absorption detector; detection wavelength: 254 nm; injection volume: 20 µL. One unit of enzyme activity is defined as the amount of enzyme required to convert 1 µmol of substrate UDP-Glu in 1 min.

### Transforming *A6180* Into *S. cerevisiae*

*Saccharomyces cerevisiae* competent cells were prepared using the Super Yeast Transformation Kit (Coolaber). The recombinant vector pESC-Leu-A6180 was transformed into *S. cerevisiae* according to the manufacturer’s instructions. Positive transformants were screened by PCR and designated as BY4742-A6180. Western blotting was used to further verify the correct expression of the protein encoded by *A6180* (Supplementary Data S1). Yeast cells were harvested after 24 h incubation in yeast extract peptone dextrose medium (YPD) (1% yeast extract, 2% peptone, and 2% dextrose) at 28°C and 160 r/min. The collected cells were frozen in liquid nitrogen and ground in a mortar with a pestle. The intracellular protein was extracted using RIPA lysis buffer (Yeasen) for western blotting. In the fermentation assay, all the *S. cerevisiae* strains were inoculated in 4 ml YPD for 18 h at 28°C and 160 r/min. One milliliter of culture suspension was used as a seed to transfer into 100 ml YPD for scale-up fermentation on different culture days (28°C 160 r/min).

### Yeast Polysaccharide Extraction and *in vitro* Immune Activity Evaluation

Yeast cells were collected by centrifuging day 1–9 of fermentation. The precipitates were washed with distilled water and freeze-dried to obtain dried cell debris. The polysaccharides of yeast cells were extracted and determined according to a previously reported method (Zhang et al., 2021). The β-glucan content of polysaccharides was determined using β-glucan assay kits provided by Megazyme International Ireland Limited (yeast and mushroom). The polysaccharide samples were hydrolyzed and analyzed by HPAEC system (Dionex ICS-2500, Dionex, Sunnyvale, CA, United States) equipped with a CarboPac™ PA20 column (Dionex, Sunnyvale, CA, United States) for monosaccharide composition testing according to the reference (Jiang et al., 2016). The generated polysaccharide solution was dialyzed (with a molecular weight of 3.0 kDa intercepted) by distilled water for 48 h and then freeze-dried. The freeze-dried sample was prepared into a 5 mg/ml original liquor with PBS (phosphate-buffered saline, pH 7.4) solution and centrifuged at 12,000 × *g* for 30 min. The supernatant was diluted to 0.5, 2.0, and 5.0 mg/ml for cell testing (final concentrations in cell culture medium were 50, 200, and 500 µg/ml, respectively). The *in vitro* immune activity of the dialyzed polysaccharide samples was studied by measuring the NO production of RAW264.7, according to the previous report (Wu et al., 2019), and the *in vitro* immunoactivity was assessed based on the amount of NO released.

### Statistical Analysis

Statistical analysis was carried out using SPSS 26.0 software (SPSS Inc., Chicago, United States). A probability level of *p* < 0.05 was set as statistical significance. Data of the NO production of RAW264.7were presented as mean ± standard deviation (SD) of at least three independent experiments.

## Results

### *A6180* Contains a Typical UGE Domain

**FIGURE 1:**
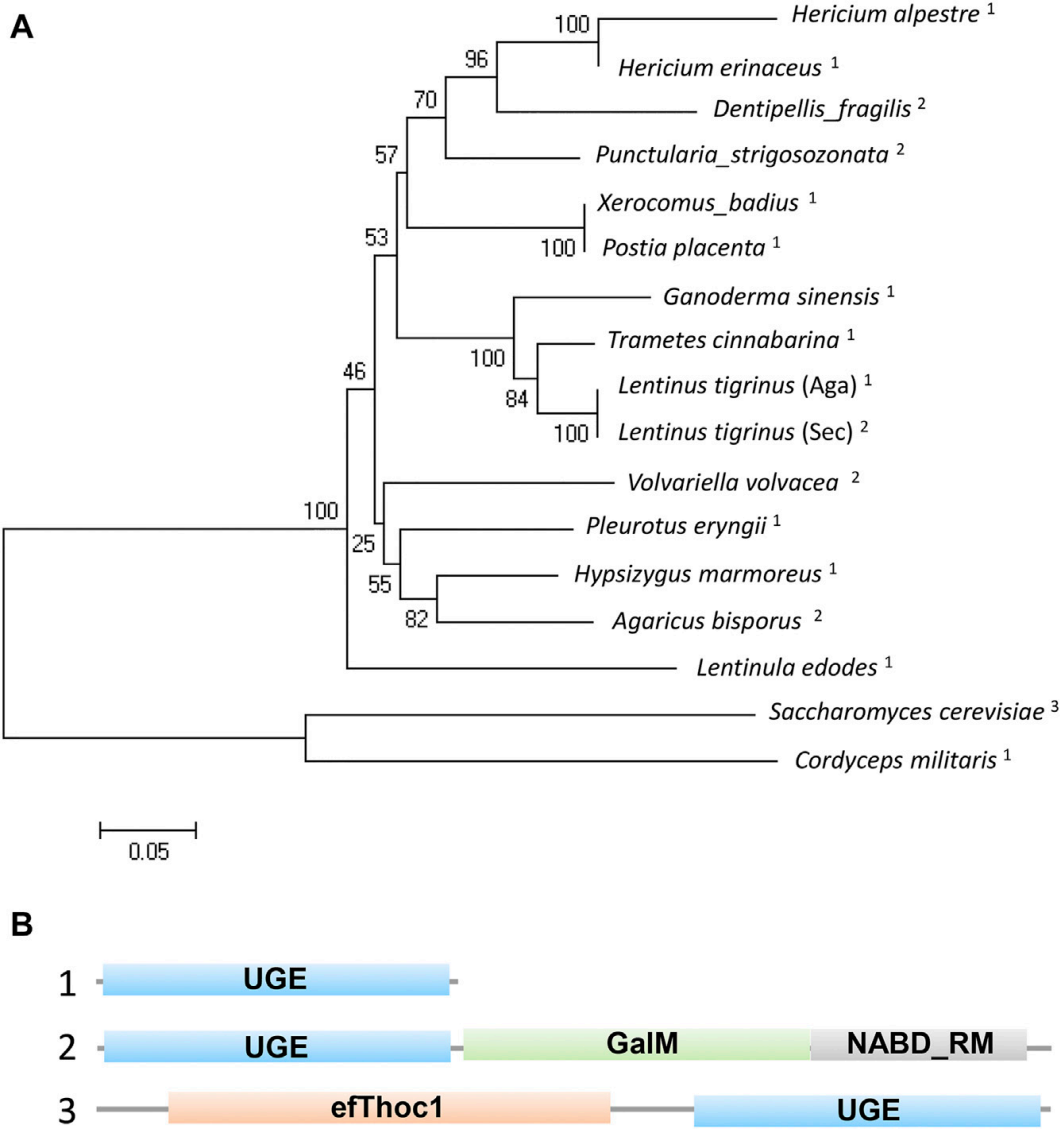
Phylogenetic and structural analysis of UDP-glucose-4-epimerases in fungi. **(A)** Phylogenetic analysis of UDP-glucose-4-epimerases of fungi including: *Hericium erinaceus* (DATA S1), *H. alpestre* (accession no. TFY78463.1), *Dentipellis_fragilis* (accession no. TFY62748.1), *Punctularia_strigosozonata* (accession no. XP_009540529.1), *Xerocomus_badius* (accession no. KAF8559888.1), *Postia_placenta* (accession no. KAF8559888.1), *Ganoderma_sinensis*_ZZ0214-1 (accession no. PIL31081.1), *Trametes_cinnabarina* (accession no. CDO77294.1), *Lentinus_tigrinus*_(Aga) (accession no. RPD64555.1), *Lentinus_tigrinus*_(Sec) (accession no. RPD82942.1), *Volvariella_volvacea*_(accession no. KAF8665304.1) *Pleurotus_eryngii*_(accession no. KDQ31371.1), *Hypsizygus_marmoreus* (accession no. RDB19506.1), *Agaricus_bisporus* (accession no. XP_006454268.1), *Lentinula_edodes* (accession no. GAW00910.1), *Saccharomyces_cerevisiae* (accession no. AJQ11874.1:), and *Cordyceps_militaris* (accession no. XP_006672787.1). A neighbor-joining tree was built using MEGA5.0 and the bootstrap method with 1000 replicates. The superscript numbers represent three types of orthologs shown in B. **(B)** The structural functional domain analysis of UDP-glucose-4-epimerases. 1) Typical UGE with unique functional domain. 2) A yeast UGE containing N-terminal epimerase domain and a C-terminal mutarotase domain. 3) An exclusive UGE to basidiomycetes containing N-terminal THO complex subunit 1 transcription elongation factor domain and C-terminal epimerase domain.

**FIGURE 2:**
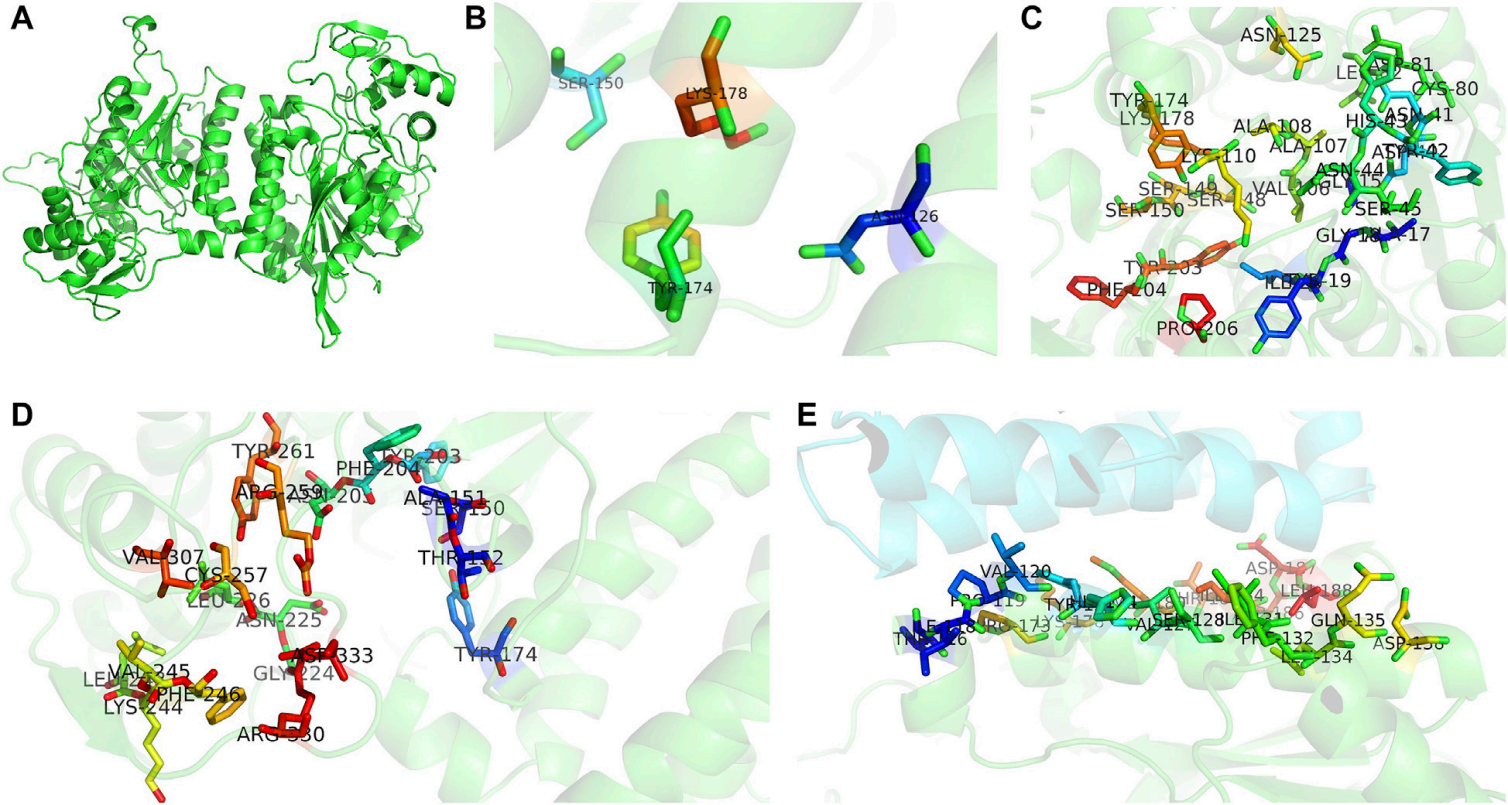
Three-dimensional structures of UDP-glucose-4-epimerases in *H. erinaceus*. **(A)** Homodimer was developed by homology modeling. **(B)** Active sites (N126, S150, Y174, K178) were illustrated in colored sticks. **(C)** NAD binding sites of *H. erinaceus* UGE were shown in colored sticks (G16, A18, G19, Y20, I21, C80, D81, L82, V106, A107, A108, K110, N125, S148, S149, S150, Y174, K178, Y203, F204, and P206). **(D)** Homology modeling of substrate binding sites (S150, A151,T152, Y174, Y203, F204, N205, G222, N225, L226, L243, K244, V245, F246, C257, R259, Y260, and V307). **(E)** Monomer interactions caused by peptide binding sites in homodimer interface (T116, I118, P119, V120, Y123, H124, V127, S128, I131, F132, L134, Q135, D138, P173, K176, M180, T183. I184, D186. D187, and L188). All models were generated by PyMOL.

To understand the functions of the protein encoded by *A6180*, a neighbor-joining phylogenetic tree was established to analyze the evolutionary relationships of fungi, including Ascomycetes and Basidiomycetes. The results showed that *H. erinaceus* was close to the fungus *H. alpestre* (Figure 1A). Intriguingly, the ortholog in *Dentipellis fragilis* was also closely related to *A6180*, although it was incorporated with an N-terminal THO complex subunit 1 transcription elongation factor domain and a C-terminal epimrase domain (Figure 1B). In *S. cerevisiae*, the ortholog protein Gal10P contains a galactose mutarotase domain (Figure 1B). Thus, the orthologs of UDP-glucose-4-epimerases in fungi are classified into three forms with distinct protein structures (Figure 1B). Based on sequence alignment in the SWISS-MODLE server (https://swissmodel.expasy.org/), the crystal structure of UDP-glucose-4-epimerases of *Burkholderia pseudomallei* (PDB ID: 3enk.1) was chosen as the template, and the tertiary structure of *A6180* was modeled using the SWISS-MODLE server (Figure 2). Combined with the prediction of conserved domains on NCBI, *A6180* was predicted to be a homodimeric UDP-glucose-4-epimerases catalyzing the NAD-dependent interconversion of UDP-galactose and UDP-glucose **(** Figure 2A **)**. It has an N-form catalytic tetrad composed of residues N126, S150, Y174, and K178 (Figure 2B). Twenty-one residues (G16, A18, G19, Y20, I21, C80, D81, L82, V106, A107, A108, K110, N125, S148, S149, S150, Y174, K178, Y203, F204, and P206) were predicted as NAD binding sites (Figure 2C) and eighteen residues were substrate binding sites (S150, A151, T152, Y174, Y203, F204, N205, G222, N225, L226, L243, K244, V245, F246, C257, R259, Y260, and V307) **(** Figure 2D **)**. These regions were so close that some of the residues overlapped, such as S150, Y174, and Y178. In the intermediate region of the homodimer, polypeptide binding motif (constituted by T116, I118, P119, V120, Y123, H124, V127, S128, I131, F132, L134, Q135, D138, P173, K176, M180, and T183. I184, D186. D187, and L188) were located in the α-helix (Figure 2E). This indicated that the monomers interacted to form homodimers.

### *A6180* Is Highly Expressed in *E. coli*

**FIGURE 3:**
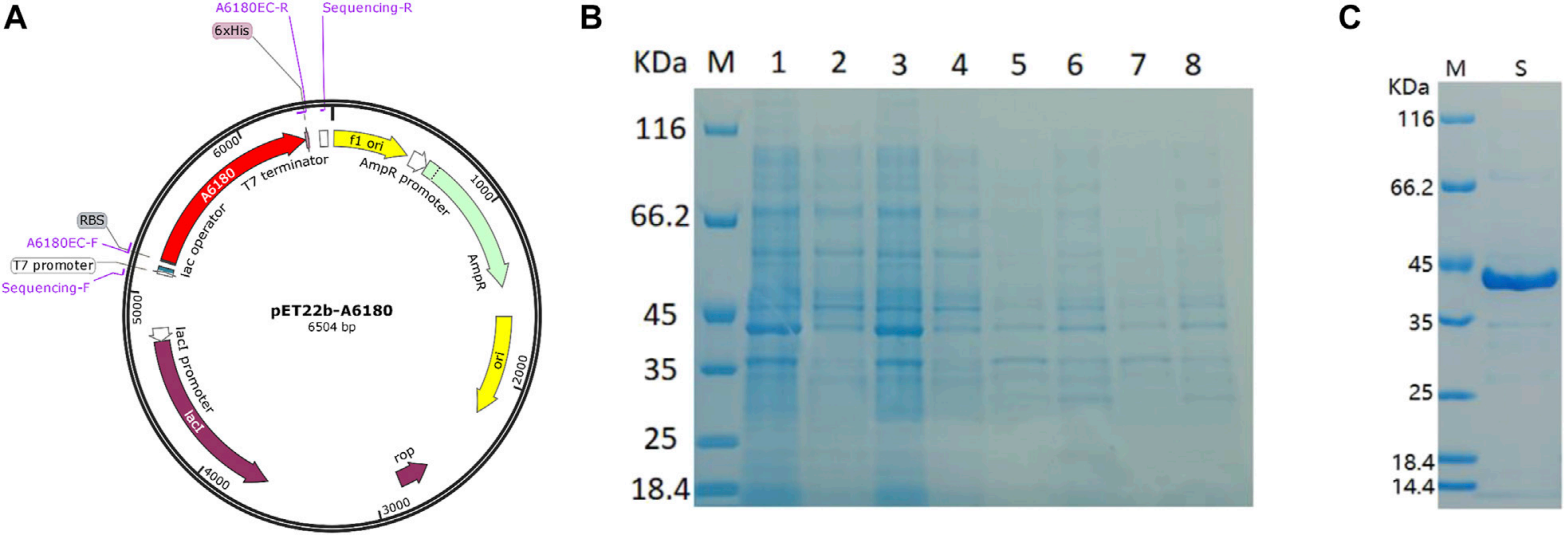
Heterologous expression of A6180 in *E. coli*. **(A)** Schematic diagram of the construction of recombinant overexpression vector. **(B)** SDS-PAGE analysis of the protein expression of positive transformants under the four inductive expression conditions. M: protein marker; 1: 37°C 1.0 mmol/L IPTG induced precipitation; 2: 37°C 1.0 mmol/L IPTG induced supernatant sample; 3: 37°C 0.2 mmol/L IPTG induced precipitation sample; 4: 37°C 0.2 mmol/L IPTG induced supernatant sample; 5: 15°C 1.0 mmol/L IPTG induced precipitation sample; 6: 15°C 1.0 supernatant sample after induction of mmol/L IPTG; 7: precipitation sample after induction of 0.2 mmol/L IPTG at 15°C; 8: sample of supernatant after induction of 0.2 mmol/L IPTG at 15°C. **(C)** Purified recombinant protein expressed in *E. coli*. M: marker; S: Purified protein.

The *E. coli* heterologous expression system is a reliable tool for characterizing protein function. Thus, *A6180* fragments were ligated into the pET22b (+) vector for protein expression. A schematic of the recombinant overexpression vector construction is shown in Figure 3A. The selected positive clones were cultured under various culture conditions. Crude extracts of cultured cell debris were verified by SDS-PAGE. The full-length recombinant putative UGE had a predicted molecular weight of 41.7 kD with 379 amino acids. SDS–PAGE analysis indicated that recombinant UGE was expressed under all the test conditions. Among these, the highest yield of recombinant protein was observed in precipitates and supernatants when the clones were induced by 1.0 mmol/L IPTG at 37°C (Figure 3B). Although most of the recombinant proteins existed as inclusion bodies in the precipitate under these conditions, the recombinant proteins in the supernatant were also the highest of all the tested conditions. Thus, *E. coli* cells were harvested from 2 L of culture suspension after 4 h of induction with 1.0 mmol/L IPTG at 37°C. Finally, the SDS-PAGE purified samples of recombinant UGE protein were analyzed using Quantity One gel analysis software, which showed that the target protein reached 95% purity (Figure 3C).

### Enzyme Activity Characteristics Show *A6180* Is a Real UGE

**FIGURE 4:**
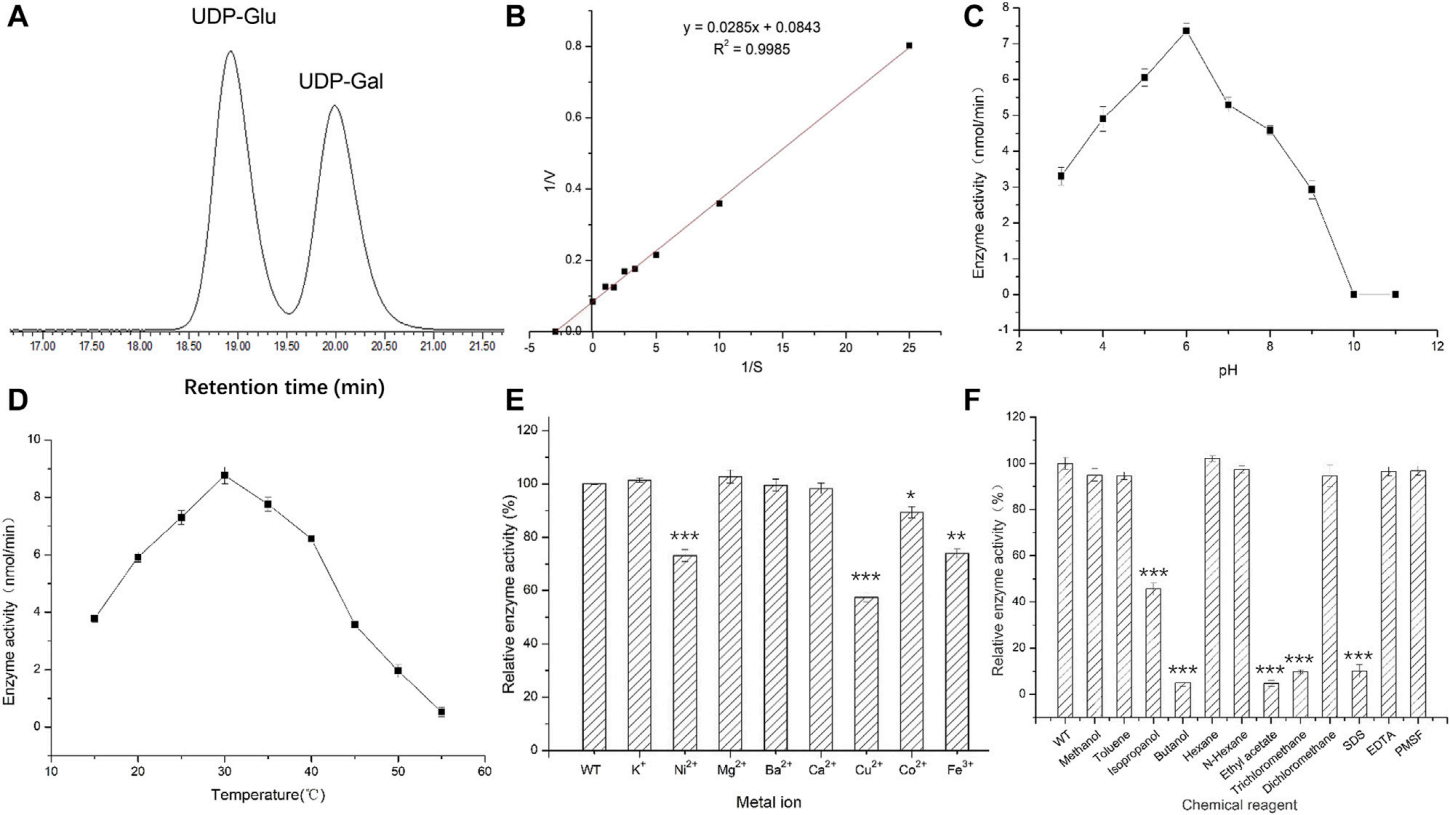
Activities of recombinant protein expressed in *E. coli*. **(A)** High performance liquid chromatography detection separation UDP-Glu and UDP-Gal. Peak 1: UDP-Glu; Peak 2: UDP-Gal. **(B)** Double reciprocal graph. **(C)** Optimal pH for activity. **(D)** Optimal temperature for activity. **(E)** The effect of metal ions on enzyme activity. **(F)** The effect of chemical reagents on enzyme activity.

The optimum chromatographic conditions were selected for the detection and separation of UDP-Glu and UDP-Gal by screening the mobile phase ratio and flow rate conditions of HPLC. As shown in Figure 4A, the absorption peaks of UDP-Glu and UDP-Gal standards appeared at 18.90 min (UDP-Glu) and 20.10 min (UDP-Gal). This indicated that the two standards could be effectively separated under the tested conditions (Supplementary Figure S1). The enzymatic reaction rate of UGE was measured for different concentrations of UDP-Glu substrate. The double inverse equation (Figure 4B) showed that the *V*_*m*_ of the target protein to UDP-Glu is 11.86 mmol/min and *K*_*m*_ is 0.34 mM. This suggests that *A6180* encodes a real UGE with the activity of converting UDP-Glu into UDP-Gal.

Under the reaction conditions of T = 35°C, the enzymatic activity of UGE protein showed an initial increasing trend and then a decreasing trend with the increase in pH, and had the highest enzymatic activity at pH 6, while the enzymatic activity was basically lost when the pH was 10.0 and 11.0, respectively (Figure 4C) (Supplementary Figure S2). This indicates that the change in pH affects the rate of enzymatic reaction by affecting the dissociation of the enzyme active center and the substrate of UGE protein, while at pH 10.0 and 11.0, the hyperalkaline state denatures the enzyme protein and thus loses its enzymatic activity.

Under the optimum pH condition for enzymatic hydrolysis, the enzyme activity first increased and then decreased with increasing temperature, and the UGE protein had the highest enzyme activity at a temperature of 30°C, but the enzyme activity decreased rapidly in the range of 35–50°C, and reached 0 at 50°C. However, when the temperature was higher than the optimum temperature, the protein gradually denatured and inactivated the enzyme, resulting in a significant decrease in enzyme activity (Figure 4D) (Supplementary Figure S3).

The general culture conditions for the mycelium of *H. erinaceus* were 26°C and natural pH medium (pH 5.8), and the results of the study also showed that the optimum pH and optimum temperature of UGE were closer to the culture conditions.

### Effects of Metal Ions and Organic Reagents on UGE Enzyme Activity

The addition of the same concentration of several metal ions at pH 6 and 30°C produced different effects on enzyme activity. Among them, K^+^ and Mg^2+^ had 2.8 and 4% enhancement effects on enzyme activity, while Ni^2+^, Cu^2+^, Co^2+^, and Fe^3+^ showed different degrees of inhibition of enzyme activity, with Cu^2+^ having the most inhibitory effect, reducing the enzyme activity to 58% of the original activity. In contrast, Ba^2+^ and Ca^2+^ had no effect on enzyme activity. This could be attributed to the metal ions combining with the sparse group, sulfur group, or amino group in the target enzyme protein molecule, thus affecting the structure of the active center of the enzyme protein molecule and leading to a reduction in enzyme activity (Figure 4E) (Supplementary Figure S4).

The different chemical reagents added to the enzyme reaction system at pH 6 and 30°C produced different degrees of inhibition of enzyme activity, with isopropanol, *n*-butanol, ethyl acetate, and trichloromethane, which showed the strongest inhibition of enzyme activity. The reaction enhanced the contact between organic solvents and water molecules through oscillation, resulting in the removal of the hydrophilic residues surrounding the surface of the enzyme protein molecules, causing changes in the spatial configuration of the protein thus reducing the enzyme activity to different degrees. The strong electrostatic interaction between SDS as an anionic surfactant and the enzyme molecule caused a change in enzyme conformation, which led to a significant decrease in enzyme activity (Guo et al., 2006) (Figure 4F).

### *A6180* Expressed in *S. cerevisiae* Constitutively

**FIGURE 5:**
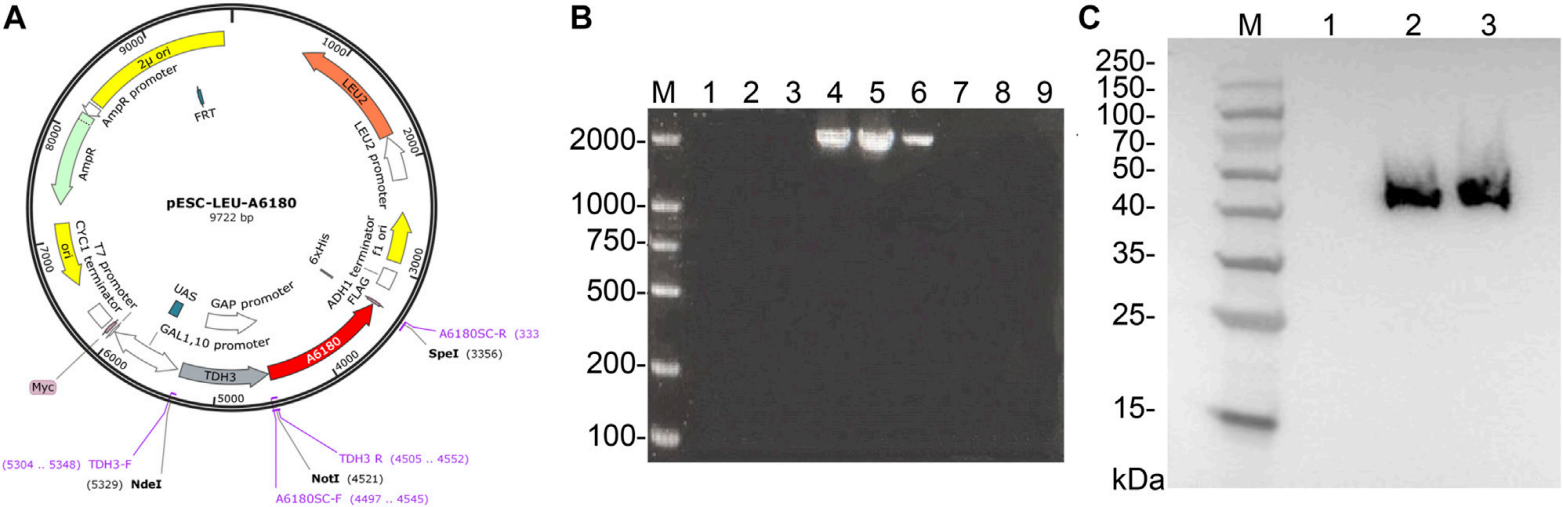
Expression of A6180 in yeast. **(A)** Schematic diagram of the construction of recombinant overexpression vector pESC-Leu-A6180. **(B)** Verification for positive transformants of yeast. M: D2000 Marker; 1–3: blank controls; 4–9: transformants 1–6. **(C)** Western blot results of positive transformants. M: Prestained protein Marker 10–180 kDa; 1: Parent strain. 2–3: Positive transformants.

A schematic diagram of the recombinant overexpression vector is shown in Figure 5A. Since the GAL1, GAL10 promoter of the pESC-Leu overexpression vector was repressed in the presence of glucose in the culture medium, the *TDH3* strong promoter of *S. cerevisiae* BY4742, which is not repressed by glucose, was added between the GAL1 and GAL10 promoters and the A6180 target gene, thus enabling stable and efficient expression of the *A6180* target gene in *S. cerevisiae* BY4742. Moreover, a 6×His protein tag coding sequence was fused to the 3′ terminal of *A6180* for Western blot analysis using the responding monoclonal antibody.

PCR was performed on nine randomly selected single colonies, and the results are shown in Figure 5B. According to the previous primer design, it can be seen that the results of lanes 4, 5, and 6 are consistent with the expected size and brightness, so the single colonies corresponding to lanes 4, 5, and 6 can be identified as positive transformant single colonies BY4742-A6180.

The total protein of the positive transformant BY4742-A6180 was verified by western blotting, and the results are shown in Figure 5C. A clear band at the size of 42 kDa consisted with the size of the target protein expressed by the *A6180* gene predicted by Expasy (https://web.expasy.org/protparam/), which can be determined that the target protein, was normally induced to be expressed in the positive transformant BY4742-A6180 induction group.

### Effect of Gene *A6180* Transformation on Physicochemical Properties of *S. cerevisiae* Polysaccharides

**FIGURE 6:**
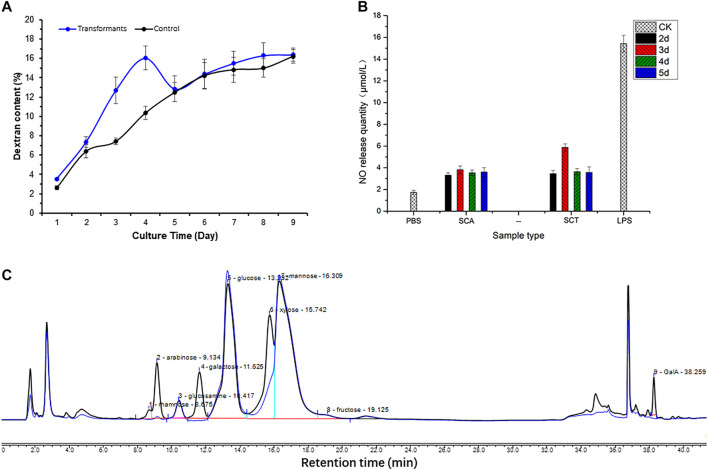
Effect on polysaccharide after expressing A6180 in yeast. **(A)** The trend of glucan in *S. cerevisiae* control group and transformation group. The result of the control group is a blue curve and the result of the transformation group is a black curve. **(B)** Monosaccharide composition results of *S. cerevisiae* control group and transformation group on day 3. The result of the control group can be seen in the blue curve, and the result of the transformation group is present in the black curve. **(C)** Immune activity of polysaccharides from *S. cerevisiae* control group and transformation group. PBS (phosphate-buffered saline): blank control; SCT: polysaccharides from *S. cerevisiae* control group; SCA: polysaccharides from *S. cerevisiae* transformation group; LPS (Lipopolysaccharides): positive control.

The total dextran of yeast polysaccharides in the original and transformed strains is shown in Figure 6A, with the blue line for the original strain (control group) and the black line for the transformed strain. The dextran content of the control group showed an increasing trend in the first 4 days, while there was a decreasing trend on the fifth day, but it slowly increased and stabilized during the sixth to ninth days. The total dextran content in the transformed and control groups was similar after cultivation for 5 days, but there was an obvious decrease in the transformed strain on the third and fourth days. This indicated that the *A6180* gene became functional on the third and fourth days, and was consistent with the bioinformatics analysis of its function as a UDP-glucose-4-epimerase (EC:5.1.3.2). *The A6180* gene was transformed into yeast by converting UDP-Glu to UDP-Gal, which in turn led to the reduction in the amount of glucan synthesis precursor substance (UDP-Glu) and consequently to a reduction in the total amount of glucan on days three and four in the transformed group compared to the control group.

Cells were collected to determine the monosaccharide composition of polysaccharide after 1–9 days of culture. After comparing the monosaccharide composition of yeast polysaccharide produced on each day, only the monosaccharide composition of the third day changed to a larger extent, and the monosaccharide composition of yeast polysaccharide on the other days remained the same (Figure 6B). The results of the monosaccharide composition data of the third day are shown in Table 2, which shows that a variety of new monosaccharides appeared in the transformation group compared with the control group. Yeast polysaccharides, usually, contain only glucose and mannose and a small amount of fructose, as shown in Table 2, and the monosaccharide composition of the polysaccharides produced by the yeast transformed with the A6180 gene contained four new monosaccharides (rhamnose, galactose, xylose, and galacturonic acid) which accounted for 0.55, 9.24, 11.83, and 1.54% of the total sugars, respectively. The percentage of arabinose increased by 3.1% and the percentage of glucosamine, glucose, mannose, and fructose decreased by 0.29, 7.88, 16.87, and 1.23%, respectively.

**TABLE 2:** The proportion of monosaccharide composition in *S. cerevisiae* and its transformant on day 3.

Monosaccharide type	Monosaccharide in control group	Monosaccharide in transformed group (%)	Increased monosaccharides in transformed group	Reduced monosaccharides in transformed group
Rhamnose	N.D^a^	0.55	0.55%	N.A^b^
Arabinose	0.15%	3.25	3.10%	N.A.
Glucosamine	0.82%	0.53	N.A.	0.29%
Galactose	N.D	9.24	9.24%	N.A.
Glucose	33.50%	25.62	N.A.	7.88%
Xylose	N.D.	11.83	11.83%	N.A.
Mannose	61.81%	44.94	N.A.	16.87%
Fructose	3.72%	2.49	N.A.	1.23%
GalA	N.D.	1.54	1.54%	N.A.

a N.D: not detected.

b N.A: not application.

Due to the overexpression of the *A6180* gene in the transformed group of *S. cerevisiae* BY4742-A6180, a new target protein was produced to participate in the polysaccharide synthesis pathway in *S. cerevisiae* cells. In turn, a new conversion pathway from glucose to galactose emerged, resulting in the production of galactose products that did not exist in the transformant strain and accounted for 9.24% of the total sugars. The increase in galactose content in the transformed group led to the production of galacturonic acid which also appeared in the transformed group. However, the target protein did not have an efficient catalytic function in the galactose-to-galacturonic acid pathway, resulting in an increase of galacturonic acid by 1.54%. Protein function prediction of the *A6180* gene by the Protein Family Data Bank (http://pfam.xfam.org/) Pfam showed that it also functions as a GDP-mannose 4,6 dehydratase (PF16363) (EC: 4.2.1.47), which converts GDP-α-D-mannose to GDP-4-dehydro-α-D-rhamnose, corroborating the appearance of rhamnose in the monosaccharide composition of the transformed yeast polysaccharide. The results also showed a small amount of rhamnose produced in polysaccharides of the Brewer’s yeast BY4742-A6180 transformation group, which did not appear in the Brewer’s yeast BY4742 control group, although not as significant as the elevation of galactose. Moreover, PF16363 domain is also contained in UDP-xylose synthase which converts UDP-glucuronic acid into UDP-xylose (Borg et al., 2021).

Compared with the control group, the composition of glucose, mannose, fructose, and glucosamine was reduced to different degrees in the transformed group. This could be attributed to the protein expressed by the *A6180* gene promoting the conversion of glucose to galactose and galacturonic acid, leading to a significant decrease in the conversion of glucose to mannose, fructose, and glucosamine. Overexpression of the *A6180* gene promoted the simultaneous conversion of glucose as a reaction substrate to multiple monosaccharide conversions, leading to an increase in the consumption of glucose, which in turn led to a 7.88% decrease in the total sugar percentage of glucose.

### *In vitro* Bioactivity of Polysaccharides From the *S. cerevisiae* BY4742-A6180

Enhancing macrophage activity *in vitro* is one way to evaluate the immune activity of polysaccharide fractions (Hitschler and Boles, 2019). After the pre-experiment of the *in vitro* immunoreactivity by determining the NO production of RAW264.7 cells treated with the *S. cerevisiae* polysaccharide on the third day at different concentrations, the highest activity of the sample was found at 500 μg/ml concentration. The 500 μg/ml concentration, therefore, was chosen to continue the immunoreactivity assay of the polysaccharide obtained from *S. cerevisiae* BY4742-A6180 transformation strain and control strain cultivated on days 2–5. The results showed that the polysaccharide activity in the transformed group increased by 71.8% on the third day compared to the control group, and there was no significant change after 4 days incubation (Figure 6C). This is also consistent with the previous results for monosaccharide composition, in which only the third day of the transformation group showed a significant change in monosaccharide composition. This indicates that the activity of yeast polysaccharides is closely related to their structure, and is especially related to the composition of its monosaccharide. The transformation of A6180 into yeast changed the structure of the yeast polysaccharides and further changed the activity of its polysaccharide.

**FIGURE 7:**
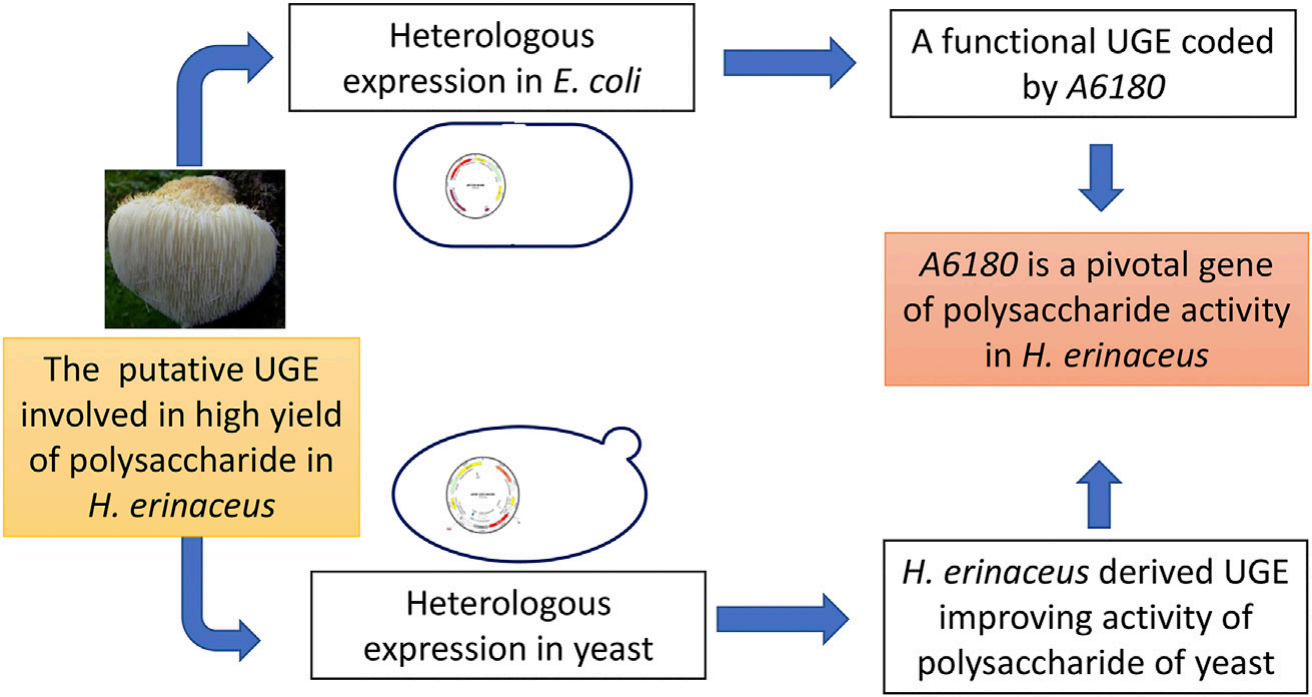
Schematic diagram of this study.

In this study, heterologous expression of *A6180* in *E. coli* and yeast confirmed that UGE encoded by *A6180* is involved in polysaccharide production by *H. erinaceus* (Figure 7). In particular, the expression results in yeast indicated that UGE derived from *H. erinaceus* could change the composition of fungal polysaccharides and increase their activity. In the future, it is promising to use it to engineer strains for producing polysaccharides with high activity.

## Discussion

Most of the current research on UDP-glucose-4-epimerases is limited to model species, including *E. coli* (Zhu et al., 2019), *Aspergillus* (Lee et al., 2014; Park et al., 2014), and *Arabidopsis thaliana* (Barber et al., 2006), rather than macrofungi. In our previous study, a predicted UDP-glucose-4-epimerases could be involved in the high yield of high-bioactivity polysaccharides in an *H. erinaceus* mutant. To date, there have been limited reports on the function of UDP-glucose-4-epimerases in macrofungi. In the present study, the enzymatic characteristics of the purified UGE (heterologously expressed in *E. coli*) showed that the optimum conditions were consistent with the cultivation conditions of *H. erinaceus*. Moreover, the heterologous expression of UGE in *S. cerevisiae* also indicates that UGE participates in the synthesis of polysaccharides.

The general culture conditions for the mycelium of *H. erinaceus* were 26°C and natural pH medium (approximately 5.8), and the results of the study showed that the optimum pH and optimum temperature of UGE were closer to the culture conditions. The optimum pH and temperature of UGE from oyster (Song et al., 2018), *E. coli* (Guo et al., 2006) were 8.5 and 8, 16°C and 37°C, respectively. This indicates that the reaction conditions of UGE from *H. erinaceus* are close to the optimum conditions for fungal growth, which is more suitable for practical production applications of UDP-Gal. This suggests that this UGE encoding gene could be used to engineer other fungi to produce highly active polysaccharides under optimal conditions with the highest activity.

The heterologous expression and polysaccharide properties of the *A6180* gene in *S. cerevisiae* yielded three results. First, the overall trend of glucan content in the experimental group was similar to that of the control group, with a significant decrease in glucan content on days 3 and 4, which tentatively demonstrated that the *A6180* gene functions on days 3 and 4; and was found to have the ability to convert glucose into other monosaccharides. UDP-glucose-4-epimerase (EC:5.1.3.2) converts UDP-α-D-glucose to UDP-α-D-galactose, corroborating the appearance of galactose in the monosaccharide composition results. It also functions as GDP-mannose 4,6 dehydratase (EC: 4.2.1.47), which converts GDP-α-D-mannose to GDP-4-dehydro-α-D-rhamnose, corroborating the appearance of rhamnose in the monosaccharide composition results. As shown by the monosaccharide composition results, it can also promote the production of xylose, and it accounts for a larger proportion of monosaccharide conversion (11.83%). The polysaccharide composition of *S. cerevisiae* is generally considered to be mainly composed of glucan and mannan. The analysis of the results of our control group is consistent with the previous report (Free, 2013). The *A6180* gene is the only difference between the transformed group and the control group. Therefore, we speculate that *A6180* coded a protein with multi-function besides the isomerization between different hexoses. This speculation is similar to that of a previously reported bifunctional UGE. It catalyzes the isomerization between a variety of UDP-sugars, including UDP-hexose and UDP-pentose (Schäper et al., 2019). In addition, the possible activities of the conserved functional domains contained in A6180 include GDP-mannose 4,6-dehydratase (Li et al., 2021), UDP-glucuronate 4-epimerase (Gauttam et al., 2021), and UDP-glucuronate decarboxylase (Woo et al., 2019), and so on. This versatile A6180 that may cause the dramatic change in the composition and proportions of yeast polysaccharides.

In the budding yeast *S. cerevisiae*, Gal10p contains both galactose mutarotase (mutarotase) and UDP-galactose-4-epimerase (referred to as epimerase) (Majumdar et al., 2004). This dual activity appears to be unique to *S. cerevisiae* and other yeasts such as *Kluyveromyces fragilis*, *K. lactis*, and *Pachysolen tannophilus* (Brahma and Bhattacharyya, 2004). It is not usual to see two non-sequential enzymatic activities encoded in the same protein, and it is not clear why the two activities are linked this way in yeasts. Previous reports have indicated that this biofunctional protein would have the advantage of sequestering galactose 1-phosphate, which is toxic to both yeasts and mammals (Tsakiris et al., 2002; Scott and Timson, 2007). However, this study showed that the expression of UGE with a single function did not cause toxicity in yeast (Scott and Timson, 2007). Moreover, large amounts of galactose were detected in the polysaccharides. Therefore, we hypothesize that the bifunctional GAL10P in yeast is responsible for the absence of galactose in wild-type yeast polysaccharides (Lozančić et al., 2021). However, it is difficult to explain the special structure of basidiomycetes. Research is yet to be carried out on the N-terminal THO complex subunit 1 transcription elongation factor domain in fungi. In humans, it functions in the cotranscriptional recruitment of mRNA to export proteins to the nascent transcript (Luo et al., 2012).

In our previous study, *A6180* was speculated to be related to polysaccharide production. However, we found that heterologous expression in yeast was not significantly related to polysaccharide yield. This may be related to the fact that we are only heterologously expressed instead of replacing the endogenous UGE gene in yeast. However, in some plant UGE functional studies, it is related to the production of polysaccharides. In *Brassica rapa*, BrUGE1 was cloned and introduced into the genome of wild type rice (Gopum) using the *Agrobacterium*-mediated transformation method. Agronomic trait evaluation of the transgenic plants under optimal field conditions revealed enriched biomass production, particularly in panicle length, number of productive tillers, number of spikelets per panicle, filled spikelets, and polysaccharide content (Guevara et al., 2014; Abdula et al., 2016). In addition, our results reveal that UGE is not only related to polysaccharide production, but also to the structure and activity of polysaccharides. It is important to conduct in-depth research on the functions of UGE.

## Data Availability

The original contributions presented in the study are included in the article/Supplementary Material, further inquiries can be directed to the corresponding author.
