# Supplementary material for: Characterization and Heterologous Expression of UDP-Glucose 4-Epimerase From a Hericium erinaceus Mutant with High Polysaccharide Production
Source: Front Bioeng Biotechnol. 2021 Nov 25;9:796278. doi: 10.3389/fbioe.2021.796278 (PMC8655778; doi:10.3389/fbioe.2021.796278)
Supplement: Supplementary file 1 [file DataSheet1.docx]

Supplementary Material

# Supplementary Data

**Supplementary Data 1:** coding sequence of *A6180*

> A6180

ATGGCTGTCGCTGATACCTCTCTTAAACGTGTTTTGGTGACGGGTGGTGCTGGGTACATCGGTTCCCATGTCATTTACGCATTACAGAAGACAAGGCGTTACAAAGTCATCTGTCTTGACAACTACCATAATTCGCAGCCGAAGGCCTTGACACGCCTCGAGCAGATCGCCACCGACGCGCTCCCGGAGGGTGCCACCGCCGACGAAAAGGCGTCCGCAGTCATAGACGTGCACAAGTGCGATCTTACCCAGCCCGAGCAGATCCGAGCAGTCTTCAAGAAGTACGGCAAGGGCGGAATATGGGGAATCATCCACGTCGCGGCCTACAAAGCTGTTGGAGAATCAACAGAGATACCCGTTACCTATTACCACAATAATGTTTCCGCTACCATATTCCTCCTCCAAGTAATGGATGACTTCGACTGCACACGATTCGTATATTCCTCCTCCGCCACCGTCTACGGTACCCCGCCAAAAGTACCCATTCCCGAGTCTACTCGTCTTCAGGCCGACAGCCCTTACGGCAAGAGCAAGGTGATGGCGGAGACGATCATTGATGATTTGACTCACGCCCAACCCACAAGATGGCGAGCTATCTCCCTGCGATACTTCAACCCCGCGGGCGCTCACCCCTCTGGTCTCATCGGTGAGGATCCCCTGGGCCGACCTGGGAATCTGCTGCCCTTGCTGGCCCAGATGGCCGTCGGCCGTGTGAAGGATCCCGTTCTGAAAGTCTTCGGCAACGACTATCCCACCCCAGACGGAACGTGCGTCCGCGACTATCTGCACGTCCTCGACCTGGCCGAGGGCCACCTGCTTGCTCTGGACGCGCTCGCGCCCGAGTCGAAGCTGTTCGACAACTGCCCCACCGAGGCGCGCTACAAGGCGTACAACCTCGGCAGGGGCCAGGGCTCCAGTGTCCTGCAGATCGTCGAGGCCATGCGCAAGGCGACCGGATTCGACTACAAGACCGAGATCGTCGGCAGGAGACGGGGCGATGTCCCAGATCTCACCGCGGACCCGACGCTCGCCGAGAAGGAGCTGGGCTTCAAGGCGAAGCAGGACCTCGAGACCGCCTGCAGGGACCTGTGGAACTGGCAGACGAAGAACCCGAATGGATACGATACCGAGTCGAAGTGA

# Supplementary Figures


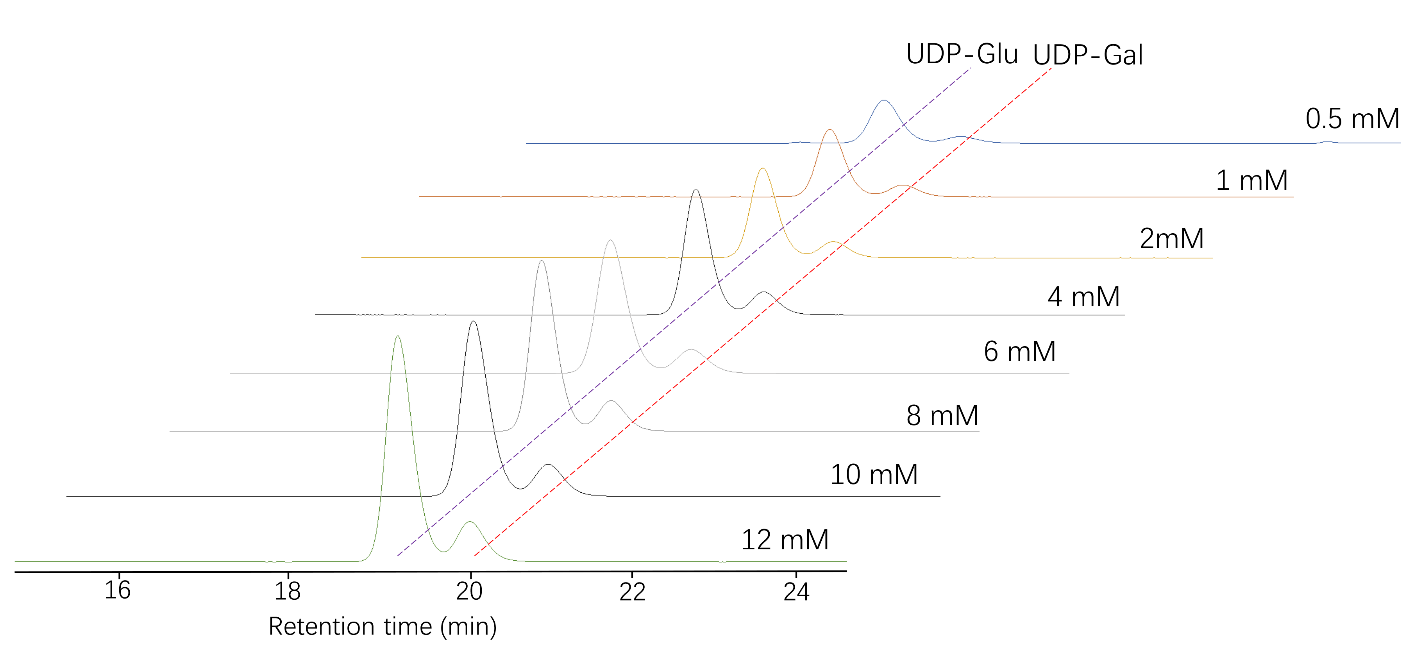


**Supplementary Figure 1.** HPLC analysis of catalytic activity of recombinant UGE coded by *A6180*. Different concentrations (0.5 mM ~ 12 mM) of UDP-Glu were loaded into the reaction. UDP-Gal production was analyzed by HPLC.


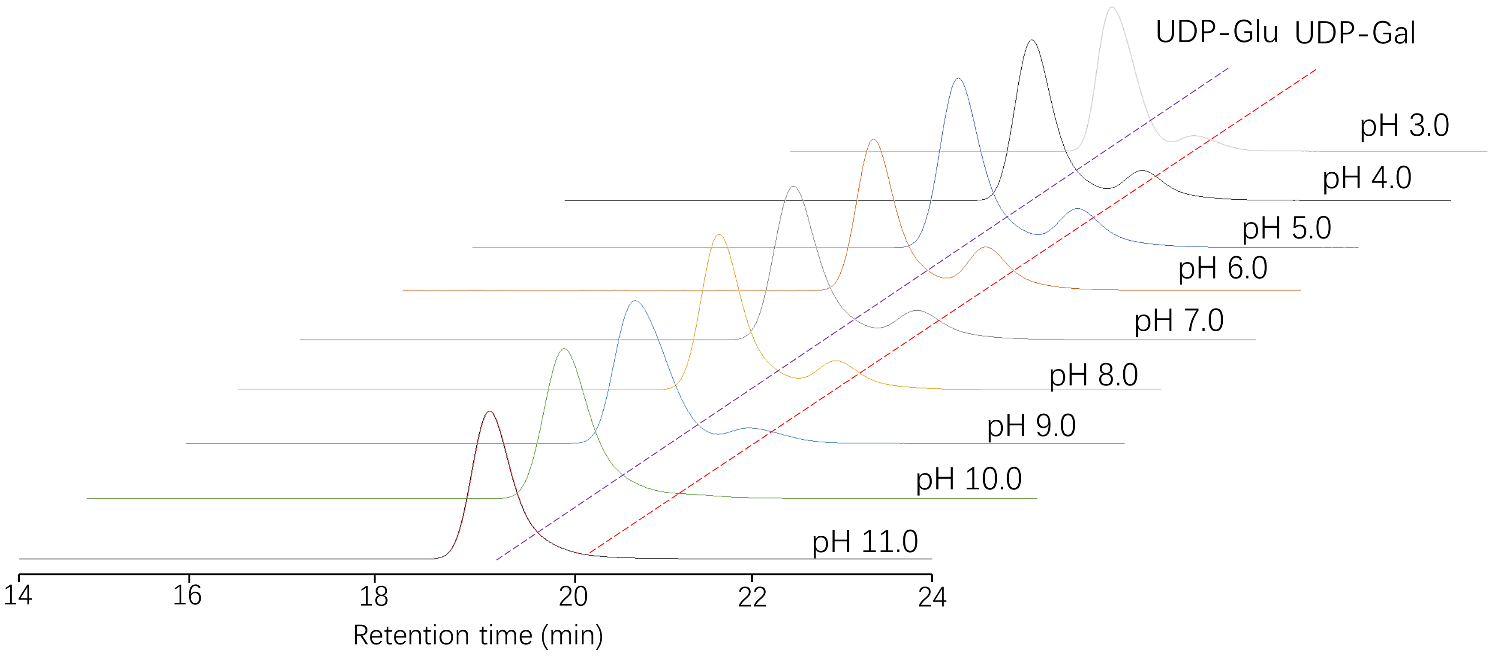


**Supplementary Figure 2.** HPLC analysis of effect of pH on catalytic activity of recombinant UGE coded by *A6180*. Different reaction buffers with pH 3.0 ~ 11.0 were used to screening the optimal pH of recombinant UGE.


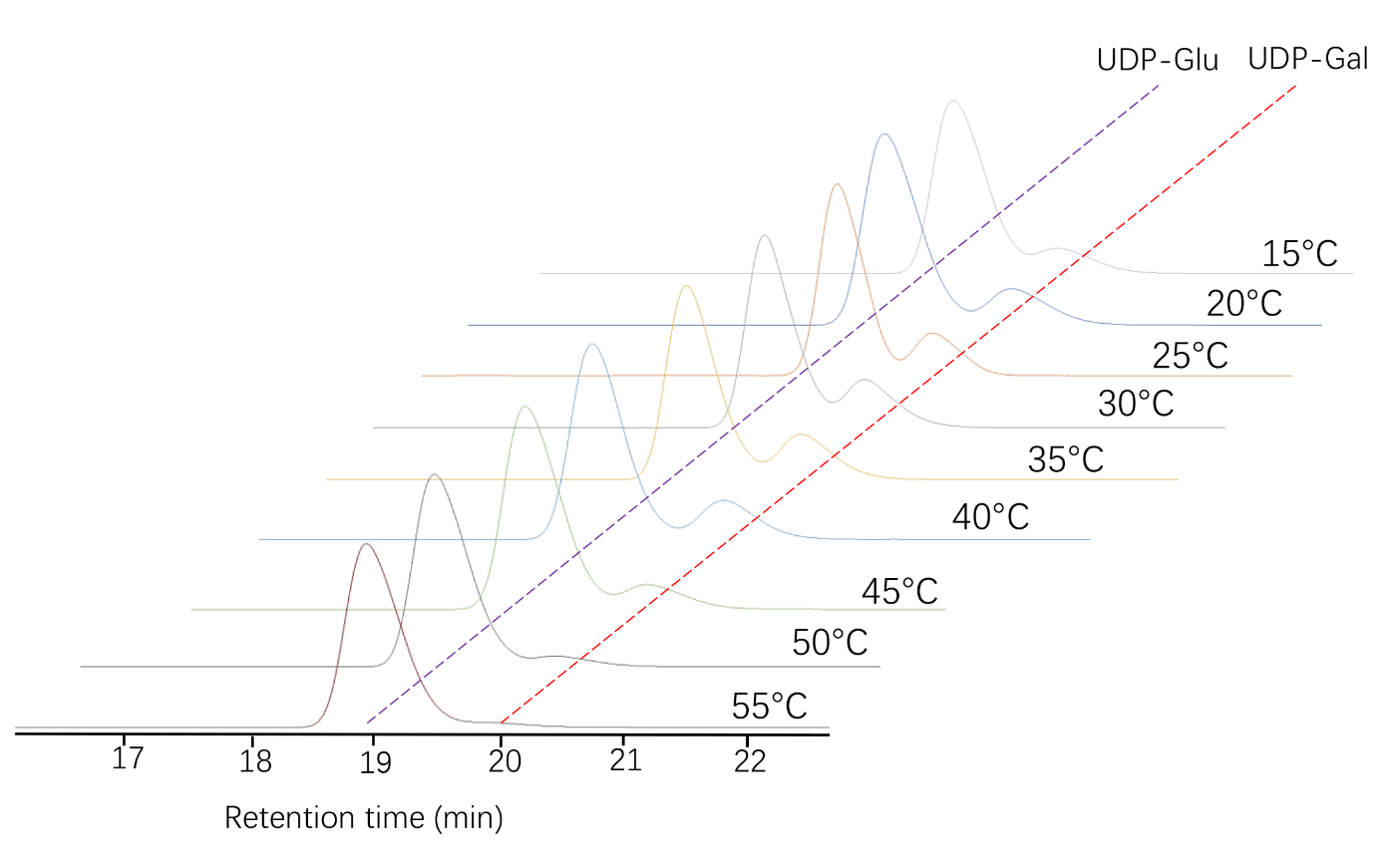


**Supplementary Figure 3.** HPLC analysis of effect of temperature on catalytic activity of recombinant UGE coded by *A6180*. Catalytic reactions are carried out at different temperatures (15°C ~ 55°C). UDP-Gal production was analyzed by HPLC.


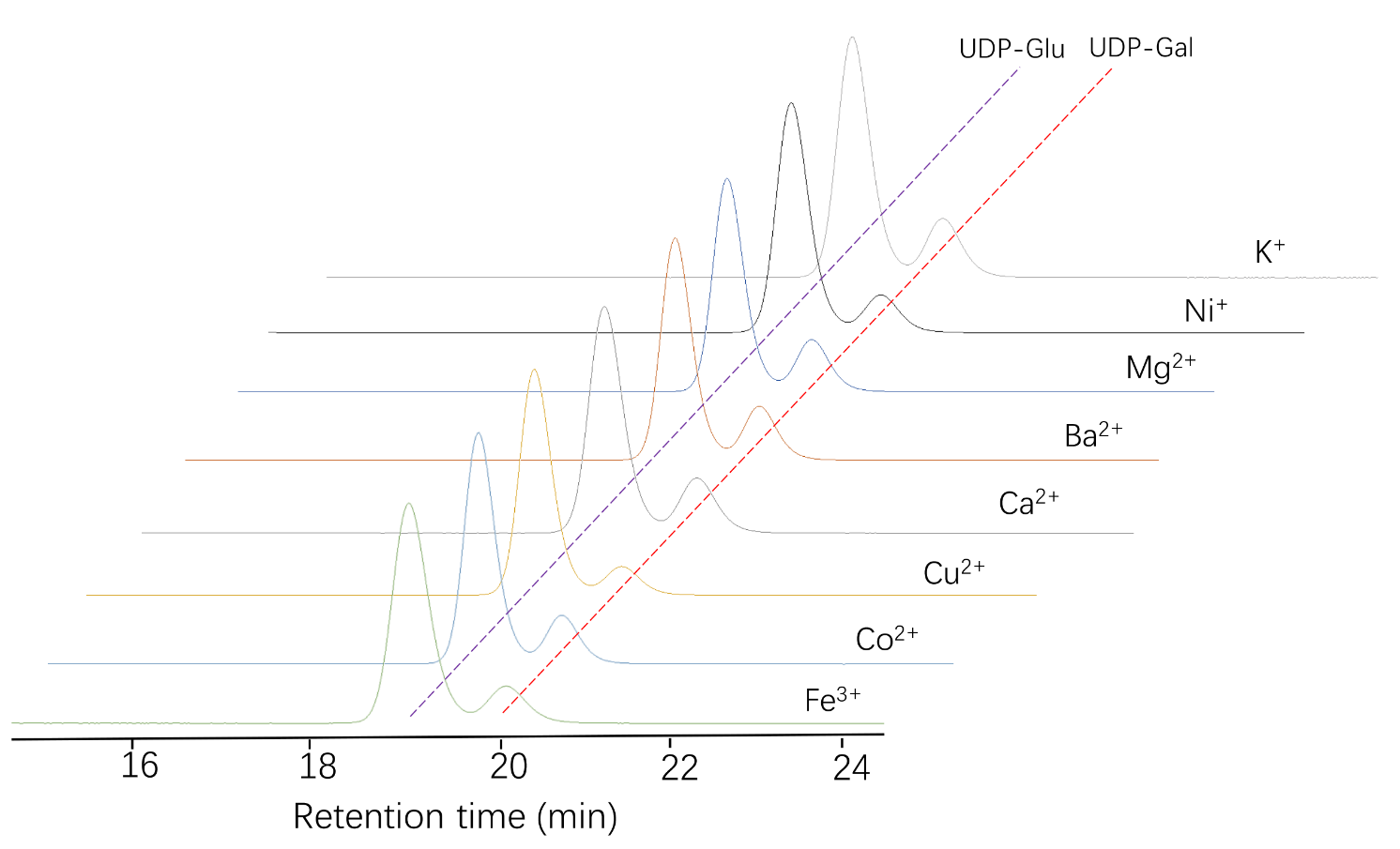


**Supplementary Figure 4.** HPLC analysis of effect of cations on catalytic activity of recombinant UGE coded by *A6180*. Catalytic reactions are carried out with 2 mM cations (K^+^, Ni^2+^, Mg^2+^, Cu^2+^, Co^2+^, and Fe^3+^) UDP-Gal production was analyzed by HPLC.
